# Effects of loneliness on short video addiction among college students: the chain mediating role of social support and physical activity

**DOI:** 10.3389/fpubh.2024.1484117

**Published:** 2024-11-12

**Authors:** Zhe Zhao, Yali Kou

**Affiliations:** ^1^Department of Physical Education, Kunsan National University, Gunsan, Republic of Korea; ^2^School of Marxism, Shangqiu Normal University, Shangqiu, China

**Keywords:** college students, loneliness, short video addiction, social support, physical activity

## Abstract

Loneliness is a common public health problem that affects physical and mental health. Prior research has demonstrated a connection between internet addiction and loneliness. Short video addiction is a novel internet addiction. It is necessary to study the potential psychological mechanisms between loneliness and short video addiction. This study investigated the associations between loneliness and short video addiction, as well as the mediating roles played by social support and physical activity.

**Methods:**

A sample of 388 college students was selected, and the questionnaires included the Loneliness Scale Short Version, the Short Video Addiction Scale, the Social Support Scale, and the Physical Activity Scale. The data were analyzed using SPSS for correlation analysis and PROCESS macros for mediation effect analysis.

**Results:**

(1) Loneliness significantly positively affected short video addiction. (2) The association between loneliness and short video addiction was independently mediated by social support. (3) Physical activity independently mediated loneliness and short video addiction. (4) Social support and physical activity play a chain mediating role in the association between loneliness and short video addiction. Our research improves the literature on loneliness and short video addiction, enhances comprehension of the impacts, and offers college students effective ways to combat the addiction.

## 1 Introduction

Since the birth of short videos, short video apps such as TikTok have proliferated worldwide. Short video apps have quickly become one of the most essential mobile apps for people’s lives, entertainment, and socialization. China had 1.012 billion short video consumers in December 2022; among them, the proportion of teenagers who had viewed short videos was 65.6%, while the percentage of active users reached 20% (1). The youth group represented by college students represents one of the most common consumers of short videos. Moderate use of short video applications can make people’s lives more exciting and convenient, whereas excessive and uncontrolled use can have serious adverse effects (2). Short video addiction involves the excessive use of short video apps and may be a type of internet addiction (3, 4). Researchers have shown that the college student population has higher-than-average levels of short video addiction (5). Similar to internet-addictive behaviors, short video addictive behaviors can cause physical and emotional harm such as attention disorders, sleep disorders, and loneliness (6–8). Interestingly, mind stream experience and cognitive lock-in make consumers want to use short video apps even after experiencing negative consequences (9).

Although digital technology has created social media platforms like YouTube and TikTok, which have enriched the social life of college students, loneliness among college students is still severe (10). The incidence of loneliness in the college student population is as high as 60.2% (11). The increase in daily smartphone use among adolescents has made them feel lonelier and more anxious (12). Research has revealed a worrisome connection between loneliness and internet addiction (13). Loneliness and internet addiction levels are positively correlated in a moderate way (14). As loneliness increases, internet addiction increases (15). Short video addiction is a new internet addiction, and the relationship between loneliness and short video addiction is unclear. The aim of this study was to determine the effect of loneliness on short video addiction and analyze the mediating roles of social support and physical activity.

## 2 Literature review

### 2.1 Loneliness and short video addiction

Short video addiction refers to overindulgence in short video-mediated activities, which is uncontrollable and leads to significant physiological, psychological, and social impairments in individuals (16). Short video apps’ tailored big-data recommendation methods create a closed-loop interaction between TikTok addiction and algorithmic optimization, worsening users’ addiction (17). Loneliness is the discrepancy between a person’s desired degree of social ties and the level of social relationships obtained (18). Loneliness is a common phenomenon in the adolescent population. As the internet and smartphones have continued to develop, more research has linked loneliness to internet and smartphone addiction (19, 20). People experiencing social phobia or loneliness may overuse smartphones and other online technologies (21). Loneliness leads to escapism to some extent, and escapism leads to TikTok addiction (22). Therefore, loneliness may also be an essential factor influencing short video addiction. Hypothesis H1: Loneliness can significantly and positively predict short video addiction.

### 2.2 The mediating role of social support

Social support refers to how interpersonal relationships may buffer a person from stressful situations (23). People often actively seek social networks to obtain social support to avoid loneliness and fulfill the need for social interaction (24). Research has shown that increased loneliness among college students of different birth cohorts is associated with decreased perceived social support (primarily objective social support) (25). According to an additional study, social support influences the associations among loneliness, anxiety, depression, and physical symptoms (26). With the increasing popularity of the internet and smartphones in the college population, college students’ access to social support has gradually been categorized into online and offline forms. Online social support may harm mental health, but offline support may help to mitigate this issue (27). Research shows that offline social support is adversely connected with internet addiction, while online social support is positively correlated with internet addiction (28). Smartphone addiction can be reduced by realistic social support (29). Recent research has also indicated that offline social support negatively predicts short video addiction (30). Hypothesis H2: Social support independently mediates the association between loneliness and short video addiction.

### 2.3 The mediating role of physical activity

Due to their academic requirements, college students often sit for long periods and generally lack physical activity (31). Research has shown that loneliness may reduce physical activity (32). A longitudinal study showed that loneliness predicted lower odds of physical activity for a period of up to 2 years, with a greater likelihood of shifting from physical activity to inactivity (33). Adolescent loneliness and physical exercise are negatively correlated (10). Research has shown that for sedentary students, the frequent use of social media is associated with a lower likelihood of vigorous daily exercise (34). Students who lacked physical activity had higher total scores and frequencies of internet addiction than did students who were regularly physically active (35). A previous study found that physical activity levels are directly and negatively correlated with internet-addictive behaviors (36). Hypothesis H3: Physical activity mediates the association between loneliness and short video addiction.

### 2.4 The chain mediating effect of social support and physical activity

Most studies favorably connect social support with teenage physical activity (37). Social support, including encouragement, role modeling, and logistical help, encourages teenagers’ physical activity (38). Studies have demonstrated that students have good attitudes toward physical activity and rely primarily on social support to change their behavior (39). Social support and sociability can moderate or modulate the relationship between physical activity and loneliness (40). Loneliness also reduces social motivation to engage in physical activity (41). When peer ties are weaker, mobile phone addiction negatively impacts physical activity (42). Poor physical activity increases the degree of smartphone addiction risk (43). Adolescents who lack physical activity are more likely to be problematic internet users (44). According to the theory of compensating internet use, when a subject is experiencing hardship and suffering from psychological problems, they may shift the focus of their life to the smartphone to maintain self-esteem, escape pain, and dissipate stress (45). Lonely college students may have less social support and physical activity, so they spend much of their time using short video apps to relieve loneliness and obtain social support, which leads to addiction. Hypothesis 4: Social support and physical activity play a chain mediating role in the association between loneliness and short video addiction.

## 3 Materials and methods

### 3.1 Participants

The survey for this study was performed from June–July 2024. The study included 420 first-year to junior college students from a central Chinese university. This study used the online questionnaire platform Wenjuanxing. Several school teachers provided the university students with the website’s QR code in order to fill out the questionnaire. Online informed consent was displayed on the questionnaire homepage, and the students could choose to give their consent before proceeding to the next step; if they did not consent, they could stop completing the questionnaire at any time. This online questionnaire was conducted anonymously, voluntarily, and confidentially. After eliminating invalid surveys, we collected valid data from 388 students. The participants’ average age was 19.95 (SD = 1.02) years. The sample included 121 (31.2%) boys and 267 (68.8%) girls. There were 124 (32%) first-year students, 211 (54.4%) sophomores, and 53 (13.6%) juniors.

### 3.2 Measures

#### 3.2.1 Loneliness scale (ULS-8)

The UCLA Loneliness Scale (ULS-8) was used in this study, developed by Hays and DiMatteo (46). A four-point Likert scale (1 = never, 4 = always) has 8 items. The scale has six positively ordered “lonely” items and two negatively ordered “nonlonely” items, with the positively stated items scored in reverse order. The scale is rated on a scale of 8–32, with higher scores indicating a greater degree of loneliness. Cronbach’s alpha was 0.81 in this study.

#### 3.2.2 Short video addiction scale

The College Student Short Video Addiction Scale by Qin (47) consists of 14 questions in 4 areas: withdrawal, loss of control, avoidance and ineffectiveness. We used a 5-point Likert scale ranging from 1 (strongly disagree) to 5 (strongly agree). Stronger total scores suggest a stronger potential to become addicted to short videos. Cronbach’s alpha was 0.93 in this study.

#### 3.2.3 Social support rating scale

The SSRS developed by Xiao was utilized in this study to assess perceived social support (48). The scale comprises 10 entries, encompassing three aspects: objective support (3 entries), subjective support (4 entries), and support utilization (3 entries). The total scores range from 12 to 66. Greater levels of support are indicated by higher scores. In this study, Cronbach’s alpha was 0.78.

#### 3.2.4 Physical activity rating scale (PARS-3)

Physical activity was tested via the PARS-3, revised by Liang (49). The scale examines physical activity in three ways: intensity, time, and frequency of participation in physical activity. Exercise amount = intensity×(time-1) × frequency. Each entry is scored from 1 to 5. The total score ranges from 0 to 100. The Cronbach’s alpha was 0.70 in this study.

### 3.3 Data analysis

Descriptive and Spearman’s correlation analyses were conducted via SPSS 26.0. For the mediation analysis, Hayes’ PROCESS macro program in SPSS was used. We used Harman’s one-way test to test for common method bias. The results revealed 14 factors with a characteristic root >1. The variation explained by the 1st factor was 18.57%, which was lower than the critical value of 40%. Therefore, there was no significant common method bias in this study.

## 4 Results

### 4.1 Description and correlation

The results of the correlation analysis between the main variables are shown in Table 1. College students’ loneliness was negatively correlated with social support and physical activity (*r* = −0.495, *p* < 0.01; *r* = −0.292, *p* < 0.01) and positively correlated with short video addiction (*r* = 0.507, *p* < 0.01); short video addiction was negatively correlated with social support and physical activity (*r* = −0.372, *p* < 0.01; *r* = −0.341, *p* < 0.01); and social support was positively correlated with physical activity (*r* = 0.323, *p* < 0.01).

**Table 1:** Correlation analysis between the variables.

	M	SD	Loneliness	Short video addiction	Social support	Physical activity
Loneliness	17.48	4.71	1	–	–	–
Short video addiction	39.49	12.09	0.507^**^	1	–	–
Social support	35.44	7.07	−0.495^**^	−0.372^**^	1	–
Physical activity	6.32	2.72	−0.292^**^	−0.341^**^	0.323^**^	1

**p < 0.01.

### 4.2 Analysis of the mediating effect

According to Wen and Ye’s mediation effects test methodology (50), the significant correlation between the variables indicated that the next step of mediation effects testing could be conducted. Bootstrap-based mediation effects were tested via Hayes’ SPSS Macro Tools Model 6 (51). The results of the regression analyses (Table 2), controlling for age, sex, and grade level, were as follows. Loneliness positively predicted short video addiction (*β* = 0.489, *p* < 0.001), negatively predicted social support (*β* = −0.484, *p* < 0.001), and negatively predicted physical activity (*β* = −0.161, *p* < 0.01). Social support was a significant positive predictor of physical activity (*β* = 0.203, *p* < 0.001). After adding social support and physical activity to the regression equation, short video addiction was negatively predicted by physical activity (*β* = −0.166, *p* < 0.001) and social support (*β* = −0.119, *p* < 0.05) and positively predicted by loneliness (*β* = 0.388, *p* < 0.001).

**Table 2:** Regression analysis between the variables.

Regression equation		Overall fit index			Significance of regression coefficient	
Result variable	Predictive variable	R	R2	F	*β*	*t*
Short video addiction	Gender	0.529	0.279	37.134	0.148	3.321^*^
	Age				0.061	1.141
	Grade				−0.073	−1.385
	Loneliness				0.489	11.203^***^
Social support	Gender	0.517	0.267	34.929	−0.092	−2.060^*^
	Age				−0.006	−0.102
	Grade				−0.112	−2.091^*^
	Loneliness				−0.484	−10.990^***^
Physical activity	Gender	0.444	0.197	18.783	−0.264	−5.585^***^
	Age				0.024	0.430
	Grade				−0.009	−0.161
	Loneliness				−0.161	−3.048^**^
	Social support				0.203	3.788^***^
Short video addiction	Gender	0.565	0.319	29.715	0.090	1.979^*^
	Age				0.064	1.231
	Grade				−0.092	−1.772
	Loneliness				0.388	7.855^***^
	Social support				−0.119	−2.383^*^
	Physical activity				−0.166	−3.513^***^

*p < 0.05, **p < 0.01, ***p < 0.001.

**Figure 1:**
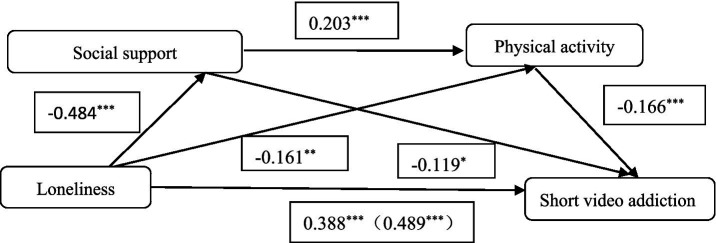
Pathway map of short video addiction affecting loneliness. *p < 0.05, **p < 0.01, ***p < 0.001.

We further tested the mediating effect (Table 3; Figure 1). With a total mediating effect value of 0.101, social support and physical activity mediated 20.65% of the total effect of loneliness on short video addiction (0.489).This mediating effect consists of three paths: first, loneliness → social support → short video addiction, with a 0.058 mediating effect value (11.86%); second, loneliness → physical activity → short video addiction, with a 0.027 mediating effect value (5.52%). Third, loneliness → social support → physical activity → short video addiction, with a 0.016 mediating effect value (3.27%).

**Table 3:** Mediation effect tests according to Bootstrap.

Benefit type	Effect value	BootSE	Bootstrap 95% CI		Proportion of relative effect
			Boot LLCI	Boot ULCI	
Indirect effect1	0.058	0.028	0.003	0.111	11.86%
Indirect effect2	0.027	0.011	0.008	0.051	5.52%
Indirect effect3	0.016	0.007	0.006	0.032	3.27%
Total indirect effect	0.101	0.028	0.045	0.157	20.65%

## 5 Discussion

According to the study’s findings, loneliness significantly positively predicts short video addiction. Much research has established the influence of loneliness on internet addiction. According to a cross-national study, teenage compulsive internet use was substantially correlated with loneliness (52). Loneliness makes smartphone users more likely to use their smartphones addictively (53). Previous relationships between loneliness and internet addiction provide us with ample experience and evidence. Our findings imply that college students’ loneliness increases short-video addiction, which harms their physical and mental health. Therefore, for college students, loneliness is a potential risk factor for short video addiction, and we must pay attention to the prevention of loneliness among college students.

We discovered that social support plays an independent mediating role in this study, verifying Hypothesis H2. Loneliness may elevate the level of short video addiction by decreasing social support. Studies have indicated that lonely people may have low social support (54, 55). Lonely individuals usually separate themselves from social communication (56). Loneliness reduces limbic and striatal activation and functional connections between the anterior insula and occipito-parietal areas, which reduces emotional reactions to pleasant social contacts (57). Teenagers who lack access to essential social networks grow lonelier and rely too heavily on the internet to cope with their emotions (58). When people cannot obtain social support in the real world, they also get social support from the online world (59). Cognitive-behavioral models show that contextual factors such as social isolation and poor social support cause maladaptive cognitions and compulsive usage (60). Therefore, the more isolated college students feel, the lower their social support level will be, raising the level of short video addiction. Helping college students acquire more real-life social support will help alleviate loneliness’s effect on short video addiction.

Our findings suggest that physical activity mediates the association between loneliness and short video addiction. Hypothesis H3 was tested. Thus, loneliness may have led to increased levels of short video addiction by decreasing physical activity levels. Previous research has found that high levels of sedentary behavior and physical inactivity are positively associated with loneliness (61). In other words, individuals who experience high levels of loneliness tend to be less physically active. Moreover, physical inactivity leads to more internet addiction behaviors (62). As a negative experience, loneliness affects the motivation and persistence of physical activity. Loneliness can have a negative impact on college students’ physical activity, and lower physical activity levels can enhance the level of short-video addiction to a certain extent (63). For college students with high levels of loneliness, physical activity is suitable for their mental health and reduces loneliness and short video addiction.

These findings suggest that loneliness can have an impact on short video addiction through the chain-mediated effects of social support and physical activity. By reducing social support and physical exercise, loneliness may exacerbate short video addiction. Teenagers may depend more on the instant gratification of cell phones than on social interactions or future rewards because of their neurological immaturity (64). Teens with internet and smartphone addiction experience significant levels of loneliness as well as unsatisfactory social relationships (65). Baumeister et al.’s ego depletion theory (66) states that ego activity depletes psychological energy, lowering executive functioning. The theory suggests that human psychological energy is limited, self-regulation and self-control weaken when psychological energy is drained. Loneliness was found to be positively correlated with cyber laziness, with ego depletion mediating this relationship (67). Thus, loneliness may drain college students’ psychological energy, reducing their self-control in realistic social and physical activities and increasing their dependence on short video applications’ instant rewards, leading to addiction. Previous research has shown that online social support partially mediates between cell phone dependence and loneliness, and can be altered to change the level of loneliness among college students who are cell phone dependent (68). In an intervention trial, loneliness was linked to perceived social support from other physical activity course members (69). Social contact during exercise impacts social support perception (70). Therefore, raising the amount of social support and physical activity can interrupt the effects of loneliness on short video addiction. For college students, establishing positive and healthy interpersonal environments and physical activity habits can help reduce their loneliness and prevent short video addiction.

## 6 Limitations and future research

This study has several limitations. First, this study was a cross-sectional study with one-time data collection, and the prediction results did not disclose the underlying causal links. A longitudinal study is needed. Second, because the variables were measured on a self-reported questionnaire, typical problems of methodological bias may have arisen. Future research could add interviews and applications that effectively measure short video addiction. Third, due to the large number of factors affecting explanatory variables, only social support and physical activity were mediating variables in this study. Future research needs to explore other potential influences in depth.

## 7 Conclusion

The findings suggest that loneliness can significantly and positively predict short video addiction and that social support and physical activity can mediate this relationship. Loneliness can affect short video addiction through the chain mediating effect of social support and physical activity. The study indicates that obtaining more social support and increasing physical activity are effective strategies for college students to cope with short video addiction. Interventions targeting loneliness may also provide a more comprehensive solution to alleviate short video addiction. These findings are essential for the development of targeted intervention strategies for short video addiction. This study provides new insights into the association between loneliness and short video addiction and also emphasizes the critical role of social support and physical activity in this association. These findings provide insights for effectively ameliorating short video addiction among college students and have a positive effect on encouraging college students to participate in real-life social activities and physical activity.

## Data Availability

The original contributions presented in the study are included in the article/supplementary material, further inquiries can be directed to the corresponding author.
